# Selecting correct functional form in consumption function: Analysis of energy demand at household level

**DOI:** 10.1371/journal.pone.0270222

**Published:** 2022-12-22

**Authors:** Hafsa Hina, Faisal Abbas, Unbreen Qayyum

**Affiliations:** Pakistan Institute of Development Economics Islamabad, Islamabad, Pakistan; Center for International Climate and Environmental Research: CICERO, NORWAY

## Abstract

In the estimation of demand functions for energy resources, parametric econometric models of energy demand are commonly used to predict future energy needs. The functional forms commonly assumed in parametric energy demand models include linear functional forms, log-linear forms, trans-log models, and an almost ideal demand system. It is frequently debated which is the “best” functional form to employ in order to accurately represent the underlying relationships between the demand for various energy resources and explanatory variables such as energy prices, income, and other demographic factors. The study has focused on developing proper non-nested tests to compare the two demand systems, the double log model and the LA-AIDS model. C-test is used to test the validity of using the two parametric functional forms in models of residential energy demand. Cross-sectional household-level data of the Pakistan Social and Living Standards Measurement (PSLM) 2013–14 and Asian Development Bank (ADB) Asia and Pacific 2018 are used. Results indicate that the LA-AIDS model is better than the double log model. The estimated elasticity of own prices, cross-prices, and income in terms of spending, family size, and equipment are particularly important to producers and policymakers in making investment and incentive choices. A significant part of the budget for families is for electricity, natural gas, firewood, and other fuels; smaller budget shares are set down to other items such as kerosene oil, cylinder gas, and diesel. Household per capita demand for energy resources will rise over the next decade; therefore, the government needs to make progress on developing energy-saving strategies. If not addressed the issue properly, we may face energy shortages and high energy import bills.

## 1. Introduction

In empirical studies of consumption and production, selecting a functional form has always been an issue of great concern. Different functional forms often lead to very distinct estimates of elasticities [1], ultimately predicting different future values and suggesting alternative policy scenarios. The latest interest is concentrated on developing an appropriate non-nested test of the two demand systems. Elasticity estimates from different models, such as an almost ideal demand system or log-log model, are used to evaluate how price, tax, earnings, climate, and other variables could affect demand [2].

For predicting future energy requirements, parametric econometric energy demand models are widely used. The frequently assumed functional forms in parametric energy demand models include linear, log-linear, and trans-log functional forms. In linear models, different explanatory variables are supposed to have a straightforward linear fashion effect on energy demand. The dependent and explanatory variables are converted into natural logarithms in log-linear models and then regressed. From the estimated coefficient, elasticities can be easily attained. To recognize their restricted theoretical basis, linear and log-linear models are sometimes referred to as ad hoc models.

In comparison, in microeconomics theory, translog models have some foundations and are common in the literature. Examples of energy demand models such as the double log model can be seen in [3, 4] for trans-log models of energy demand in [5]. A range of other models are found in the scholarly literature but are less frequently used by practitioners, including types of Almost Ideal Demand Systems (AIDS), Symmetric Generalized McFadden (SGM), and Generalized Leontief (GL) forms. The literature in economics [2] frequently used a non-parametric bootstrapping technique to compare functional forms of linear, log-linear, and trans-log share equations with a non-parametric function. The same exercise was repeated in [6] using the same example application and data set of [2] but using an alternative Bayesian technique.

In this analysis, we are using an alternative approach developed by [7], also known as the C-test. Under the null hypothesis, the double log model for elasticity estimation is the correct functional form. This approach was used by [8] for the estimation of compensated double log demand model by deflating the income variable alone using the stones price index. The compensated form has the same right-hand side as a single-equation version of the popular linear approximation to the Almost Ideal demand model, facilitating the construction of a test for choosing between the two alternatives. This study determines these results, develops the specification test, and illustrates its application using Pakistan energy consumption data.

Energy is a major economic sector and plays a crucial role in the economic development of the country. In the past, Pakistan’s economy was faced with energy-side blockages that had restricted its growth and development. In addition to the growth of natural resources and minerals, the state is attempting to guarantee the accessibility and safety of renewable energy, petroleum and gas supply. Pakistan is gradually moving to a decarbonization system and concentrating more on renewable energy sources in accordance with the Paris Climate Agreement to reduce emission intensity [9]. The government demonstrates dedication through renewable energy sources to generate electricity. Renewables currently account for only two percent of electricity generation, although they are anticipated to rise in the coming years.

In the energy sector, energy consumption per capita is regarded to be one of the most significant indicators of economic welfare in terms of accessible energy supply. In terms of consumption among different consumer classes, the domestic sector (residential consumers) showed a tremendous increase in energy use between the fiscal year 1992 and fiscal 2006; this sector’s energy consumption grew at an annual rate of 5.4 percent (ESMAP, 2006). The consumption pattern of electricity has not changed significantly over the past year, although households ’ share of electricity consumption has risen marginally to 51 percent. This was offset by a one percent decrease in the industry’s share of energy usage. During FY 2016–17, the country’s annual consumption of petroleum products was around 26 million tons. 60.4 million barrels of crude oil were imported during July-Feb FY 2017–18, while 21.8 million barrels were extracted locally. Only 15 percent of the country’s total requirements are met by indigenous crude oil, while 85 percent are met by imports in the form of crude oil and refined petroleum products (Pakistan Economic Survey 2017–18).

Coal is known as one of the cheaper sources in terms of electricity generation cost (Rs/Kilowatt/hours). Gas is also a less expensive source as it is an economical and effective fuel compared to other oil goods; the national government began importing Liquefied natural gas (LNG) in the first quarter of 2015. In case of natural gas, the gap between supply and demand widened owing to increased demand for gas and the depletion of current sources. Natural Gas is a fuel that is clean, secure, effective, and friendly to the environment. Their indigenous supplies make up around 38 percent of the country’s complete primary energy production mix. The government is following its policies to boost indigenous gas manufacturing and import gas to satisfy the country’s growing demand for energy. The average consumption of natural gas During July-Feb 2017–18, was about 3.837 million cubic feet per day (MMCFD), including 632 (MMCFD) volumes of Regasified Liquefied Natural Gas (RLNG), compared to 3.205 (MMCFD) last year (Pakistan Economic Survey 2017–18). Pakistan has big reserves of indigenous coal estimated at over 186 billion tons that are adequate to satisfy the country’s long-term sustainable energy demands. Imports of coal have increased significantly as new coal-based power plants have been commissioned in Sahiwal and Port Qasim.

Recently, technological advancement, demands for renewable inclusion, and aging infrastructure have made energy forecasting more important for activities in the energy system. Management of energy demand is needed to allocate available resources appropriately. In Pakistan, there is a significant gap between electricity demand and supply, even though the government has done much to mitigate this. Now it is important to know whether this is a supply-side phenomenon, errors in demand measurement of the households, or forecasting issues? So, the idea is to explore the true pattern of household energy consumption by using household-level data. This study aims to test whether the double log functional form or linear approximation of the almost ideal demand system (LA-AIDS) provides sensible descriptions of the real functional connection of Pakistan’s household-level demand for fuel energy (Expenditure on different types of fuel–firewood, kerosene oil, natural gas, cylinder gas, diesel, and other fuels. The other fuels category includes household expenses on coal and other biomass fuels such as dung cakes and crop residue.), other fuels, and multiple explanatory variables. We also projected the future level of energy demand in terms of income elasticities through a simple growth model.

## 2. Review of literature

### 2.1 Studies on correct functional form

Authors [1] have constructed a parametric bootstrap test to choose between the linearized version of the Almost Ideal Demand System (FDAIDS) First-Difference and the model search in Rotterdam. It is known that parametric bootstrap tests have excellent size and performance characteristics, while low power is available for encompassing tests. The new approach was used to select between the FDAIDS and Rotterdam models for U.S. meat demand. With the parametric bootstrap, the FDAIDS was consistently rejected in favor of the Rotterdam model. Thus, the results support using the Rotterdam model for U.S. meat demand. Another drawback of the comprehensive test is that the compound model converted to a local rather than a global optimum in one instance.

The functional forms in demand for energy modeling are compared in [2]. He compared linear, log-linear, and translog share equation functional forms. Bootstrapping techniques are used in residential energy demand models to assess the validity of using the three parametric functional types. Using cross-sectional household-level information from the U.S. Labor Statistics Bureau (US BLS), consumer spending survey, and other public data sets. Based on the assumption that a non-parametric kernel regression estimator may provide an ideal, or at least better, description of the fundamental relationship between electricity consumption and a series of four prevalent independent variables, three popular parametric model specifications were screened and dismissed at ordinary significance levels. Each parametric functional forms investigated performs badly, implying that they may not be flexible enough to deliver relevant outcomes in some implementations. These findings indicate that when making judgments about the functional form of energy demand systems, caution should be undertaken.

Authors in [6] used the Bayesian method to evaluate what are the "best" functional forms to use in order to better depict the fundamental relationship between the consumption of different energy resources and explanatory variables like power prices, weather variables, earnings, and other variables in US demand for electricity. Excellently-known model choice measures including the Akaike Information Criterion (AIC) and the Bayesian Information Criterion (BIC) restrict Deviance Information Criterion (DIC) examples. They evaluate household energy consumption using cross-sectional family-level information, a DIC comparison for four excellently-known models demonstrates that almost ideal demand system and translog models are competitive to a double log model that is superior to the linear functional form in general.

Previous studies estimated the function of demand in accordance with economic theory. Most implemented flexible functional forms that depend strongly on the theory of duality. The most prominent demand systems are the generalized Leontief, translog, Rotterdam models, and the almost ideal demand system (AIDS). Their functional shapes are regionally flexible because at a specified stage they do not place priority constraints on possible elasticity. They also use adequate variables at a specified stage to estimate elasticity; moreover, local elastic functional forms frequently display small regular areas in accordance with macroeconomic theory.

### 2.2 Studies on energy demand in pakistan

Authors in [6] used data from the 1984–85 household integrated economic survey (HIES) to analyze the trend of household spending on energy consumption in Pakistan. They used the Extended Linear Expenditure System to evaluate price and income elasticity. The research findings indicated that almost all fuels were inelastic in price and income, implying for rural and urban residents respectively these were necessities. In addition to earnings and price, there are many other variables such as household size and social-economic variables that have an important effect on the consumption of household fuel but have not been included these factors in their study.

Extended Linear Expenditure System (ELES) is used by [10] and found, insignificant variation in the marginal budget shares and subsistence expenditure among all provinces except for transport in rural Baluchistan and rent in urban Punjab. The information is extracted from the 1986–88 household integrated economic survey. The model’s major weakness is that the estimated marginal budget shares are consistent with changes in income. Whereas the ELES is simple to use, it assumes additive preferences, significantly limiting the options of replacement and also excluding inferior goods.

[11] explained the status of biomass energy use in Pakistan. Moreover, accurate estimates of the use of biomass energy in separate sectors of the economy are not accessible, as in most developing countries. It has been estimated that around 65.07 billion kg of firewood production is equivalent to 22.57 million tons of oil equivalent (MTOE) and accounts for 44% of the country’s total main energy requirement. The residential home field is the leading consumer and uses up 86% of the amount of energy from biomass. The traditional cooking stoves are the main consumers of renewable energy and about 80% of the total amount they consume.

[12] reviewed Pakistan’s disaggregated power requirement (gas, electricity, and coal) over the 1972–2007 era. Their primary findings indicate that the consumption of electricity and coal is responding positively to fluctuations in real GDP per capita and the national price level would be negatively affected. Gas consumption reacts negatively to short-term real income and price modifications, but in the long-term real income has a positive impact on gas usage, while the national price stays negligible. In addition, the average price elasticity and real gas consumption income (in absolute numbers) are higher in the short run than those of electric power and oil demand. Each energy component’s variations in elasticity have important policy consequences for earnings and generating revenue.

[13] investigated the reactions of residential electricity demand to revenue modifications to help policymakers in controlling electricity demand and assessing tariff rises connected with suggested projects to increase supply while minimizing the effect on hunger. For 2003/04, they used Punjab Multiple Indicators Cluster Survey (MICS) data. The MICS has information on appliance owning, accommodation, and household features (such as number of members of the household and number of rooms in living space) and per capita income and expenditure for over 30,000 households, in addition to the required information on electricity expenditure. Accordingly, electricity demand is positively dependent on both income and ownership of the appliance. Appliance ownership has dramatically increased and almost all families have an electricity connection.

[14] forecasted complete energy usage and its elements for Pakistan up to 2020, such as household, other government, agriculture, street light, industrial and commercial sectors. From 1980 to 2011, they implemented Holt-Winter and Autoregressive Integrated Moving Average (ARIMA) models on secondary time series data to predict cumulative and part-wise consumption of electricity in Pakistan. Their findings show that demand in the domestic industry would be higher than in all other industries, and the rise in power supply would be lower than the rise in total energy usage over the predicted era.

[15] estimated energy spending and family fuel cost elasticity in Pakistan. It is depleting forests, natural gas, and other reserves of energy. They pooled three information sets (2007–08, 2010–11, and 2013–14) of Pakistan’s Social and Living Standard Measurement Survey (PSLM). They reported our data set doesn’t have market price information, and the LA-AIDS model is widely used for this type of data set because all households are assumed to have the same prices fixed for this model. In addition, the LA-AIDS model is relatively simple to assess and interpret and accurately fulfills the theorems of selection. They discovered that all kinds of fuel excluding natural gas were price inelastic at the domestic and urban household levels. Fuel expenditures elasticities for all fuels were found to be positive and between zero and one.

## 3. Methodology

### 3.1 The theoretical background of demand-side analysis

The demand of households is analyzed by using numerous models from which the most common are double log functional form and linear approximation of almost ideal demand system.

#### 3.1.1 Double log model

A logarithmic Marshallian demand system is stated as;

$${\text{lnQ}}_{i}=\alpha_{0}+\overset{n}{\underset{j=1}{\sum }}\alpha_{ij}{\text{lnp}}_{j}+\alpha_{m}\;\text{ln}\;I$$ (3.1)

where Qi is quantity demand for i^th^ commodity, pj is the prices for good j, I shows the income or expenditure on all n commodities. In double log model coefficients are directly interpreted as elasticity, therefore, αij are the own price and cross-price elasticities and αm is income elasticity.

#### 3.1.2 Compensated double log demand function

Hicksian or compensated demand elasticity easily attains from the Slutsky equation. [16] proposed the Slutsky equation in elasticity deduced from Marshallian elasticity to achieve compensated demands i.e.,

$$\alpha_{ij}=\alpha_{ij}^{*}-\omega_{j}\alpha_{m}I$$ (3.2)

Where αij is the uncompensated or Marshallian price elasticity of demand for good i with respect to Pj, α* is Hicksian or compensated price elasticity of demand for good i with respect to Pj, ωj is the budget share of good j and *α*_*m*_*l* is income elasticity of good j.

By putting Eq (3.2) in Eq (3.1) we get compensated double log model,

$${\text{lnQ}}_{i}=\alpha_{0}+\overset{n}{\underset{j=1}{\sum }}\alpha_{ij}^{*}{\text{lnp}}_{j}+\alpha_{m}\;\text{ln}(\frac{I}{P^{*}})$$ (3.3)

Where the P* is Stone price index:

$$P^{*}=p_{1}{}^{\omega 1}+p_{2}{}^{\omega 2}+p_{3}{}^{\omega 3}\ldots \;\ldots \;\ldots +p_{N}{}^{\omega N}$$ (3.4)

#### 3.1.3 Almost Ideal Demand System (AIDS)

Almost Ideal Demand System (AIDS) model is introduced by [17]. Accordingly, AIDS is obtained by using the expenditure function and price-independent generalized logarithmic (PIGLOG) preferences. For utility u and price vector p, the expenditure function is e (u, p) and the PIGLOG is described as;

lne(u,p)=(1−u)lna(p)+ulnb(p) (3.5)

Functional forms available to log a(p) and log b(p) are

$$lna\left(p\right)=\alpha o+\overset{n}{\underset{i=1}{\sum }}\alpha_{i}\;ln\;p_{i}\;+\frac{1}{2}\;\overset{n}{\underset{i=1}{\sum }}\overset{n}{\underset{j=1}{\sum }}\gamma_{ij}\;lnp_{i}\;ln\;p_{j}$$ (3.6)

$$lnb\left(p\right)=\text{ln}\;a\left(p\right)+\beta_{0}\;\Pi \;p_{i}\beta i$$ (3.7)

Finally, the almost ideal demand system is written as,

$$ln\;e\left(u,\;p\right)=\alpha_{0}+\sum \alpha_{i}lnp_{i}+\frac{1}{2}\sum \sum \gamma_{ij}lnp_{j}+u\beta_{0}\Pi pi^{\beta i}$$ (3.8)

Where parameters are αi, βi, and γij. It can be easily verified that e(u, p) is linearly homogeneous in p as long as all the demand function restrictions must hold. Budget share of good i is obtained by applying Shephard Lemma, and multiplying pi/e (u, p) as,

$$\frac{dln\;e\left(u,p\right)}{dlnp_{i}}=\frac{p_{i}q_{i}}{e\left(u,p\right)}=\omega_{i}$$ (3.9)

Where ωi is the budget share of good i. By taking the partial derivative of expenditure function in Eq (3.8) in terms of logarithmic prices yields

$$\omega_{i}=\alpha_{i}+\overset{n}{\underset{i=1}{\sum }}\;\gamma_{ij}\;ln\;p_{j}+\beta_{i}\;\text{ln}\left(\frac{x}{p}\right)$$ (3.10)

Where *γ*_*ij*_ = ^1^ (*γ*_*ij*_ + *γ*_*ij*_) and x/p is real spending on all commodity groups and *p* is an index of prices.


$$ln\;a\left(P\right)=\alpha_{0}+\sum \alpha_{k}\;ln\;p_{k}+\frac{1}{2}\;\sum \sum \gamma_{ij}\;ln\;p_{k}\;ln\;p_{j}$$ (3.11)

Eq (3.11) Shows the mixture of linear and non-linear rates and is also known as the Quadratic almost ideal demand system (QA-AIDS), but [17] linearized the Almost Ideal Demand System model by replacing the nonlinear price index with the Stone price index. By deflating the income by stone price index and imposing homogeneity restriction we get a linear almost ideal demand system (LA-AIDS) as shown below:

$$\omega_{i}=\alpha_{i}+\alpha_{i1}\;\text{ln}\left(p_{1}\right)+\cdots +\alpha_{iN}\;\text{ln}\left(p_{n}\right)+\alpha_{m}\text{ln}\left(\frac{I}{P^{*}}\right)$$ (3.12)

The restrictions on the parameters of the AIDS budget share equation. We take these in three sets such as adding up, homogeneity, and symmetry restrictions which are shown below respectively:

1.
$$\sum \alpha_{i}=1,\sum \alpha_{ij}=0\;\text{and}\;\alpha_{m}=0$$ (3.13)
2.
$$\sum \alpha_{ij}=0$$ (3.14)
3.
$$\alpha_{ij}=\alpha_{ij}$$ (3.15)

Provided above three characteristics hold the budget share equation of LA-AIDS reflects a system of demand functions adding up to total expenditure shares (∑wi = 1) are homogeneous of degree zero in prices and total expenditure combined and satisfying Slutsky symmetry. By taking the derivative of Eq (3.12) w.r.t prices we get uncompensated own price and cross-price elasticities where i = 1 for own price and i = 2…n for cross-price elasticities for a good one,

$$\in_{ij}=\frac{\alpha_{ij}}{\omega_{i}}-\frac{\alpha_{m}}{\omega_{i}}-\sigma_{ij}$$ (3.16)

When *i* = *j* then *σ*_*ij*_ = 1 which will be own-price elasticity and if *i* ≠ *j* than *σ*_*ij*_ = 0 which will be uncompensated cross-price elasticity.

For expenditure or income elasticity, we take the partial derivative of Eq (3.12) w.r.t income which yields the income elasticity,

$$\eta_{i}=1+\frac{\alpha_{m}}{w_{i}}$$ (3.17)

Where *η*_*i*_ is income elasticity and if the coefficient of logarithmic income (*α*_*m*_) is positive then income elasticity will be greater than 1, that commodity is luxury. If income elasticity is less than 1 and positive then the coefficient is negative and commodity is a necessity. The commodity is unitary elastic if *α*_*m*_ = 0.

### 3.2 Test for the correct functional form

[18] indicated that the selection of the demand model is of main interest in consumer analysis since it has a direct connection to the nature of the variables or elasticity achieved. The subjective criteria for selecting functional from is depending upon the theoretical supremacy of the model. For example, LA / AIDS is flexible in permitting but not requiring overall demand theory restrictions, whereas, the double log demand function fulfills the restrictions of the demand theory and its parameters give direct elasticities. The contribution of the present study is to encompass these two demand functions for the correct functional form through the C-test by [9].

To show that the two specifications are being tested, consider the right side of the compensated double log model in Eq (3.3) where income is deflated by stone’s price index is similar to the equation of the LA/AIDS in Eq (3.12) with the expense share of the good i as the dependent variable instead of ln Qi:

$$\omega_{i}=\alpha_{i}+\alpha_{i1}\;\text{ln}\left(p_{1}\right)+\cdots +\alpha_{iN}\;\text{ln}\left(p_{n}\right)+\alpha_{m}\text{ln}\left(\frac{I}{P^{*}}\right)$$ (3.18)

Where *ω*_*i*_ = *PiQi*/*l*.

**Hypothesis**: The double log model is correct under the null hypothesis,

$$\left(1-\lambda \right)lnQ_{i}+\lambda \omega_{i}=\alpha_{i}+\alpha_{i1}\;\text{ln}\left(p_{1}\right)+\cdots +\alpha_{iN}\;\text{ln}\left(p_{n}\right)+\alpha_{m}\;\text{ln}\left(\frac{I}{P^{*}}\right)$$ (3.19)

The outcome of the estimated λ with the variables on the right side was viewed either as a test of specification or as a test of the sufficiency of the demand system linear approximation. By rearranging the above Eq (3.19) we get

$$lnQ_{i}=\alpha_{i}+\alpha_{i1}\;\text{ln}\left(p_{1}\right)+\cdots +\alpha_{iN}\;\text{ln}\left(p_{n}\right)+\alpha_{m}\;\text{ln}\left(\frac{I}{P^{*}}\right)+\lambda \left(lnQ_{i}-\omega_{i}\right)$$ (3.20)

Now there are two interpretation problems, the first is that the estimation of λ from OLS depends on the scalability of the dependent variable, and the secondly is that the estimation of λ is associated with the error term so that the result can be biased. We substitute the two possible dependent variables with their expected values in order to solve this issue $\hat{lnQ_{i}}\;and\;\hat{\omega }$ also to avoid the singular model replace other remaining right-hand side variables with one predicted value,

$$lnQ_{i}=\alpha_{i}+\alpha_{i1}\;\text{ln}\left(p_{1}\right)+\cdots +\alpha_{iN}\;\text{ln}\left(p_{n}\right)+\alpha_{m}\;\text{ln}\left(\frac{I}{P^{*}}\right)+\lambda \left(\hat{lnQ_{i}}-\hat{\omega_{i}}\right)$$ (3.21)

$$lnQ_{i}=\hat{\;lnQ_{i}}+\lambda \left(\hat{lnQ_{i}}-\hat{\omega_{i}}\right)$$ (3.22)

The prediction error in the double log model closely resembles the C-test of the Davidson and Mackinnon: $lnQ_{i}-\hat{\;lnQ_{i}}$ the λ sign is contrary to the normal C-test sign. The second issue was the scaling of the dependent variable so that the OLS estimates move towards minimizing the total amount of the linear combination of two residual vectors of the double log equation (e1) and the share equation (e2):

$$\text{min}\;e_{3}^{,}e_{3}=\text{min}{\left(1-\lambda \right)}^{2}e_{1}^{,}e_{1}+\lambda^{2}e_{2}^{,}e_{2}+2\lambda \left(1-\lambda \right)e_{1}^{,}e_{2}$$ (3.23)

The linear combination of e1 and e2 is presented by e3 so the resulting estimate of λ is given low:

$$\hat{\lambda }=\frac{e_{1}^{,}e_{1}-e_{1}^{,}e_{2}}{e_{1}^{,}e_{1}+e_{2}^{,}e_{2}-2e_{1}^{,}e_{2}}$$ (3.24)

The above equation shows that $\hat{\lambda }$ depends upon the scaling like e_2_ changes while e1 will not change so the estimated λ will be variant to scaling. To resolve this issue, there is also another modification that instead of using the observed value directly from the share equation, one can use the shared model to predict shares and transform each expected share into a ln(Qi) prediction that results can be compared with results obtained directly from the double log model. So that’s the Davidson and Mackinnon C-test in reality. Calculated on the basis of Eq 3.25:

$$lnQ_{i}=\hat{lnQ_{i}}+\lambda \left(\hat{lnQ_{i}}-\tilde{lnQi}\right)$$ (3.25)

Thus, if the double log model was true, according to the null hypothesis $lnQ_{i}-\hat{\;lnQ_{i}}$ should not be correlated with the difference of another remaining part like $\hat{\;lnQ_{i}}-\tilde{lnQi}$. So if the calculated λ is significant as proof against the null hypothesis that the log-log model is accurate [9].

#### 3.2.1 Demand projection model

In developing countries like Pakistan policies regarding household energy demand, energy supply, production, and distribution depends on energy demand forecast so demand projections are essential for development. For demand projections, some determined estimates of income elasticity, population growth rate, and income growth rate are required. In developing countries, for energy demand, there are some problems for projection, among which most famous are fast-growing population, industrialization and changing preferences, etc. In this study, we are concerned with the demand forecast of energy at a constant rate of income and population. The simple growth model will be used for projecting the energy demand. Several researchers, including [19, 20], used this formula.

Using the following growth formula, energy consumption is estimated:

$$D_{t}=D_{0}\times P_{t}{\left(1+G\times e\right)}^{t}$$ (3.26)

Here

*D*_*t*_ = current year household demand for commodity group and t = 1, 2, 3…….n. D_0_ = base year per capita consumption of commodity group here t = 0.

Pt = current year population (million) Using a simple compounding formula, the ADB data set allows us to project the future level of Pakistan’s population from 2015 to 2030.


$$\text{Population}\;\text{future}=\text{Population}\;\text{present}*{\left(1+\text{g}\right)}^{\text{t}}\;\text{Where}:\;\text{g}=\text{growth}\;\text{rate}\;\text{of}\;\text{population}.\;\text{t}=\text{projected}\;\text{year}.$$

G = GDP per capita growth rate of the current year.

e = income elasticity for the particular commodity.

Because it needs fewer data and parameters, this formula is commonly used to project demand. This model utilizes several assumptions such as steady population growth, no change in taste and preferences, steady prices, and steady manufacturing technology. This study offers the 2015–2030 energy consumption predictions.

### 3.3 Commodities included in energy demand analysis

The household energy demand is categorized into two broad groups like fuel demand and other-fuels demand and electricity. The expenditure on fuels has been further disaggregated into expenditures on different types of fuel–firewood, kerosene oil, natural gas, cylinder gas, diesel, and other-fuels. The other fuels category includes household expenses on coal and other biomass fuels such as dung cakes and crop residue. Using the price of electricity, family income, family size and ownership of electrical appliances explains the household energy demand. In the case of Pakistan, the selected appliances are freezer (fzr), refrigerator (frg), air conditioner (aclor), air cooler (aclor), washing machine (wm) and computer.

We will estimate the following general form of the system equation:

$$Q_{i}=(Y_{i},P_{1}\ldots \;\ldots ,P_{7},NF_{i},DAP_{i},)$$ (3.27)

Where Qi is energy demand by the household for all explanatory variables for i = (1, 2, 3…, 7) here (1 is firewood, 2 is kerosene oil, 3 is natural gas, 4 is cylindrical gas, 5 is diesel 6 is electricity and 7 are other fuels,), P1 = price of firewood, P2 = price of kerosene oil, P3 = price of natural gas, P4 = price of cylindrical gas, P5 = price of diesel, P6 = price of electricity and P7 = price of other fuels. Y is household income, NF is family size and DAP indicates the existence of a specific appliance. For the presence of the specific appliance, the value of each category is 1 and 0 otherwise.

#### 3.3.1 Data

This study is based on the micro-level data from the 2013–14 Pakistan Social and Living Standards Measurement (PSLM). This data set consists of 17989 households as a nationally representative sample, out of which 387 (2.15 percent) households have not reported expenditures on particular variables which are used in the analysis. So the sample of 17602 (97.9 percent) households is used for analysis. Total household expenditure on energy demand is categorized into two broad groups like fuel and other-fuels expenditures and electricity. The expenditures on fuel have been further disaggregated into expenditures on different types of fuel–firewood, kerosene oil, natural gas, cylinder gas, diesel, and other-fuels. The other-fuels category includes household expenses on coal and other biomass fuels such as dung cakes and crop residue, these are the important source of energy. Income data of households is equal to the expenditures of the household and prices data will also be calculated from the expenditure. SPSS package is used to arrange the PSLM (2013–14) data set.

For demand projections at an aggregate level, the projected data on per capita GDP growth, population, and population growth rate from 2015 to 2030 are taken from the key Asia and Pacific 2018 Asian Development Bank (ADB) indicator.

### 3.4 Econometric modeling

There are two models to estimate the elasticities as double log model and LA-AIDS from which any one model will be selected on the basis of C-test,

- **Double log model**
  The compensated double log model for various energy commodities is presented below:
$$lnQ_{i}=\alpha_{i}+\gamma_{i1}lnPF_{i}+\gamma_{i2}lnPK_{i}+\gamma_{i3}lnPN_{i}+\gamma_{i4}lnPC_{i}+\gamma_{i5}lnPD_{i}+\gamma_{i6}lnPE_{i}+\gamma_{i7}lnPOF_{i}+\gamma_{i8}NF_{i}+\gamma_{i9}DAP_{i}+\beta_{i}\;\text{ln}\left(\frac{Y}{P^{*}}\right)+\in_{i}$$ (3.28)
  Where Qi energy consumption, PF is the price of firewood, PK is the price of kerosene oil, PN is the price of natural gas, PC is the price of cylindrical gas, PD is price of diesel, PE is electricity price, POF are prices of other fuels, NF is number of family members, DAP indicates the existence of specific appliances, Y is income of the household and P* is stone price index define in Eq 3.6.
- **Almost Ideal Demand System**
  The linear approximation of the almost ideal demand system (LA-AIDS) for various energy commodities is given below:
$$\omega_{i}=\alpha_{i}+\alpha_{\text{i}8}NF+\alpha_{\text{i}9}DAP+\beta_{i}(lnY-lnP_{s}\;)+\sum^{7}\;\gamma_{ij\;}lnP_{j},+\epsilon_{i},$$ (3.29)
  Where dependent variable (*ω*_*i*,*s*_) Indicates the spending share of the s-th households of ith energy commodity for *i*, *j* = 1, 2, … …, 7 (1 is firewood, 2 is kerosene oil, 3 is natural gas, 4 is cylindrical gas, 5 is diesel, 6 is electricity and 7 is other fuels. NF represents the number of family members and DAP are Appliances), *Y* describes the household’s average nominal energy expenditure and *lnP*_*s*_ is the stone price index calculated as follows:

$$\text{ln}P_{s}=\sum^{7}\;\omega_{i},\;\text{ln}p_{j},$$ (3.30)

## 4. Result and analysis

In this section, an analysis of energy demand and projections for energy demand for Pakistan is given. This chapter is divided into four sections. Section 4.1 explains descriptive statistics of important variables used in this study. Section 4.2 explains the estimated elasticities and their implications. Section 4.3 explains the correct functional form through C-test. Section 4.4 explains the energy demand and its projections for the years 2015 to 2030.

### 4.1 Descriptive statistics

The descriptive statistics of budget share and prices of Fire wood, Kerosene oil, Natural gas, Cylinder gas, Diesel, Electricity and Other fuels are presented in Table 1. It is noted that electricity is the main source of energy consumption having an average budget share of 44.94 percent among energy expenditure whereas firewood, other fuels, natural gas, cylinder gas, kerosene oil, and Diesel for generators have average budget shares of 24.77, 14.75, 11.19, 2.80, 1.18 and 0.37 percent respectively, So diesel & petrol for the generator has very small budget share percentage because this is a very expensive source of energy. Average price for firewood (Rs/kg), kerosene oil (Rs/Ltr), natural gas (Rs/MMBTU), cylinder gas (Rs/kg), diesel (Rs/Ltr), electricity (KWh) and other fuels (Rs/kg) are 8.63, 122.91, 2.56, 138.73, 118.96, 12.27 and 4.55 percent respectively. The coefficient of variation for prices of various energy commodity groups ranges between 3.71 to 234.47 percent and the largest variation is observed for the price of other fuels category. This is attributed to large differences in the price of various other fuel types such as coal, dung cakes, biomass fuels, and crop residue. The coefficient of variation is small for kerosene oil and diesel because there are almost the same prices all over Pakistan. Household size ranges from 1 to 47, the minimum size is 1 and the maximum is 47, average household size is 7 members.

**Table 1:** Descriptive statistics.

Energy commodity	Mean	Std. Deviation	Coefficient of variation
**Budget shares**			
Fire wood	24.77	31.17	125.84
Kerosene oil	1.18	5.82	491.94
Natural gas	11.19	17.89	159.89
Cylinder gas	2.80	10.74	383.41
Diesel	0.37	3.39	926.30
Electricity	44.94	27.75	61.75
Other fuels	14.75	24.41	165.46
**Price units**			
Fire wood	8.63	2.31	26.79
Kerosene oil	122.91	4.56	3.71
Natural gas	2.56	1.37	53.60
Cylinder gas	138.73	10.72	7.73
Diesel	118.96	5.08	4.27
Electricity	12.27	1.57	12.78
Other fuel	4.55	10.67	234.47
	Minimum	Maximum	Mean
**House-hold Size**			
	1	47	7

Source: Authors’ calculation based on Pakistan PSLM information (2013–14).

### 4.2 The uncompensated double-log demand model

The estimated elasticity of the uncompensated double log demand model as shown in Eq 3.1 is presented in Table 2. The system contains seven energy commodities i.e., Fire wood, Kerosene oil, Natural gas, Cylinder gas, Diesel, electricity and last for other fuels which are estimated by OLS method. Out of seventy-seven parameters of seven equations, sixty-three parameters are statistically significant (at a level of 5%) whereas one parameter is significant at a level of 10% and plausible for their corresponding variables, but only thirteen parameters are statistically insignificant (γ23, γ26, γ27, γ29, γ31, γ35, γ39, γ41, γ43, γ49, γ54, γ58, γ79,) which are not plausible for their corresponding variables.

**Table 2:** Uncompensated double-log demand model.

Parameters	Estimates	Std. Error	Parameters Estimates	Std. Error	Parameters
**α1**	7.518***	1.103	**γ45**	-1.201*	0.735
**γ11**	-1.031***	0.024	**γ46**	-0.169***	0.061
**γ12**	2.635***	0.213	**γ47**	-0.595***	0.179
**γ13**	0.493***	0.040	**γ48**	-0.023***	0.006
**γ14**	-0.178**	0.072	**γ49**	-0.023	0.109
**γ15**	-3.308***	0.301	**β4**	0.951***	0.038
**γ16**	-0.044**	0.018	**α5**	25.834***	6.663
**γ17**	-1.616***	0.077	**γ51**	-0.724**	0.292
**γ18**	0.010***	0.002	**γ52**	-2.734*	1.421
**γ19**	-0.112***	0.019	**γ53**	-0.415***	0.082
**β1**	0.908***	0.014	**γ54**	-0.754	0.535
**α2**	27.806***	6.353	**γ55**	-1.000***	0.197
**γ21**	-0.147*	0.082	**γ56**	-0.464**	0.230
**γ22**	-0.949***	0.308	**γ57**	-3.536***	0.533
**γ23**	0.110	0.147	**γ58**	0.007	0.010
**γ24**	-0.826**	0.353	**γ59**	-0.483**	0.214
**γ25**	-4.423***	1.384	**β5**	1.170***	0.100
**γ26**	0.062	0.068	**α6**	-2.536***	0.847
**γ27**	-0.331	0.297	**γ61**	0.078***	0.023
**γ28**	0.018***	0.005	**γ62**	-1.854***	0.167
**γ29**	-0.061	0.059	**γ63**	-0.499***	0.017
**β2**	0.392***	0.048	**γ64**	0.253***	0.064
**α3**	3.670*	2.088	**γ65**	0.820***	0.160
**γ31**	0.064	0.105	**γ66**	-0.083***	0.016
**γ32**	0.857*	0.455	**γ67**	1.681***	0.058
**γ33**	-0.305***	0.013	**γ68**	-0.011***	0.002
**γ34**	-0.889***	0.202	**γ69**	0.347***	0.017
**γ35**	-0.001	0.127	**β6**	0.847***	0.011
**γ36**	0.786***	0.113	**α7**	30.447***	1.988
**γ37**	-1.498***	0.071	**γ71**	0.503***	0.044
**γ38**	0.018***	0.002	**γ72**	-6.883***	0.369
**γ39**	0.018	0.030	**γ73**	-0.644***	0.077
**β3**	0.599***	0.013	**γ74**	0.389***	0.150
**α4**	17.687***	2.974	**γ75**	0.974*	0.519
**γ41**	-0.019	0.074	**γ76**	-0.662***	0.020
**γ42**	-2.322***	0.541	**γ77**	-1.312***	0.139
**γ43**	0.224	0.152	**γ78**	0.013***	0.003
**γ44**	-1.000***	0.081	**γ79**	-0.024	0.026
			**β7**	0.593***	0.020

Source: Authors’ calculation based on Pakistan PSLM information (2013–14).

***, **, and * are used for 1, 5, and 10% significance level, respectively.

All the uncompensated own price elasticity statistics have an accurate (negative) sign that a commodity’s price itself has an adverse effect on its unit demand except electricity for which own price of electricity is positive as the demand for electricity cannot be reduced due to raise in price. Fourteen elasticities out of forty-two uncompensated cross-price elasticities are positive, meaning gross substitute, and the other twenty-eight elasticities are negative, showing complementary consumer goods. All the elasticities of the estimated income are positive. Estimated income elasticity for firewood, kerosene oil, natural gas, cylinder gas, other fuels, and electricity is positive, and less than one indicates that these products are normal and necessary, but for Diesel is greater than one indicates that the luxury commodity.

#### 4.2.1 The compensated double log demand model

The estimated elasticity of the compensated log-log demand model is shown in Table 3. Where the system of equations for seven energy commodities is estimated by using OLS method. Out of seventy-seven parameters of seven equations, sixty-one parameters are statistically highly significant (at 5% significance level) and plausible for their corresponding variables, but only sixteen parameters are statistically insignificant (γ19, γ21, γ22, γ23, γ27, γ29, β2, γ31, γ35, γ39, γ46, γ56, γ58, γ59, γ75, γ68) which are not plausible for their corresponding variables. The fact that the price of a commodity itself has an adverse effect on its quantity demand is clarified by all the compensated own price elasticity estimates except electricity. Out of the forty-two compensated cross-price elasticities, twenty-two is positive, meaning gross substitute, and the other twenty are negative, indicating complementary consumer goods. The estimated income elasticity of firewood, kerosene oil, and electricity is positive but less than one indicates that these items are normal and necessary, but for natural gas and other fuels the income elasticity is positive, and greater than one shows that these are normal and luxuries items, while income elasticity for cylindrical gas and diesel is negative indicating that these items are inferior.

**Table 3:** Compensated double-log demand mode.

Parameters	Estimates	Std. Error	Parameters Estimates	Std. Error	Parameters
**1**	3.339***	1.081	**γ45**	-1.443*	0.846
**γ11**	-0.544***	0.022	**γ46**	-0.081	0.071
**γ12**	2.745***	0.208	**γ47**	2.217***	0.188
**γ13**	0.737***	0.039	**γ48**	0.034***	0.006
**γ14**	0.170**	0.070	**γ49**	0.405***	0.124
**γ15**	-3.125***	0.295	**β4**	-0.278***	0.028
**γ16**	0.143***	0.018	**α5**	28.810***	8.016
**γ17**	-0.809***	0.072	**γ51**	-1.167***	0.346
**γ18**	0.011***	0.002	**γ52**	-3.884**	1.684
**γ19**	-0.020	0.018	**γ53**	0.167*	0.096
**β1**	0.814***	0.012	**γ54**	-1.065*	0.635
**α2**	24.039***	6.715	**γ55**	-0.902***	0.234
**γ21**	0.056	0.082	**γ56**	-0.286	0.272
**γ22**	-0.383	0.321	**γ57**	1.891***	0.594
**γ23**	0.060	0.155	**γ58**	0.007	0.011
**γ24**	-0.736**	0.373	**γ59**	0.260	0.258
**γ25**	-4.188***	1.461	**β5**	-0.291***	0.101
**γ26**	0.184***	0.070	**α6**	-5.370***	0.960
**γ27**	0.280	0.307	**γ61**	0.369***	0.026
**γ28**	0.028***	0.006	**γ62**	-1.807***	0.190
**γ29**	0.016	0.062	**γ63**	-0.173***	0.019
**β2**	0.058	0.043	**γ64**	0.377***	0.073
**α3**	5.091***	1.428	**γ65**	0.811***	0.181
**γ31**	-0.048	0.072	**γ66**	0.152***	0.018
**γ32**	0.557*	0.312	**γ67**	3.580***	0.057
**γ33**	-0.339***	0.009	**γ68**	0.002	0.002
**γ34**	-1.242	0.139	**γ69**	0.480***	0.019
**γ35**	0.076*	0.087	**β6**	0.404***	0.011
**γ36**	0.606***	0.077	**α7**	23.002***	1.546
**γ37**	-1.130***	0.037	**γ71**	0.716***	0.034
**γ38**	0.007***	0.001	**γ72**	-4.775***	0.290
**γ39**	-0.015	0.020	**γ73**	-0.445***	0.060
**β3**	1.038***	0.009	**γ74**	0.291**	0.117
**α4**	27.337***	3.392	**γ75**	-0.042	0.405
**γ41**	0.362***	0.084	**γ76**	-0.515***	0.015
**γ42**	-4.716***	0.617	**γ77**	-1.033***	0.099
**γ43**	0.729***	0.174	**γ78**	-0.013***	0.002
**γ44**	-0.509***	0.091	**γ79**	-0.043**	0.020
			**β7**	1.071***	0.015

Source: Authors’ calculation based on Pakistan PSLM information (2013–14).

***, **, and * are used for 1, 5, and 10% significance level, respectively.

In Table 3, the estimated parameters γi8 & γi9 reflect the positive and significant effect of household size and appliances for electricity on energy demand.

#### 4.2.2 Estimates of LA-AIDS parameters

Table 4 presents the estimated parameters of the LA-AIDS model. Out of total seventy-seven parameters of the LA-AIDS model for seven commodities, sixty-eight parameters are statistically highly significant (at 5% significance level) and Plausible for their respective budget shares, but only nine coefficients are statistically insignificant (γ24, γ32, γ33, γ37, γ42, γ53, γ56, γ59, γ74) which are not plausible for their corresponding budget shares.

**Table 4:** LA-AIDS parameters.

Parameters	Estimates	Std. Error	Parameters Estimates	Std. Error	Parameters Estimates
**α1**	-1.964***	0.363	**γ45**	0.230***	0.024
**γ11**	-0.108***	0.010	**γ46**	0.007***	0.002
**γ12**	1.294***	0.072	**γ47**	0.206***	0.008
**γ13**	0.166***	0.007	**γ48**	0.003***	0.000
**γ14**	-0.076***	0.027	**γ49**	0.035***	0.003
**γ15**	-0.370***	0.068	**β4**	-0.107***	0.001
**γ16**	0.040***	0.007	**α5**	0.077*	0.046
**γ17**	-0.753***	0.022	**γ51**	-0.002*	0.001
**γ18**	0.008***	0.001	**γ52**	0.093***	0.009
**γ19**	-0.111***	0.007	**γ53**	0.001	0.001
**β1**	0.009**	0.004	**γ54**	0.007**	0.003
**α2**	0.641**	0.030	**γ55**	-0.128***	0.009
**γ21**	0.003***	0.001	**γ56**	0.001	0.001
**γ22**	-0.117***	0.006	**γ57**	0.032***	0.003
**γ23**	0.012***	0.001	**γ58**	0.000***	0.000
**γ24**	0.002	0.002	**γ59**	0.001	0.001
**γ25**	-0.015***	0.006	**β5**	-0.004***	0.001
**γ26**	0.008***	0.001	**α6**	-1.364***	0.278
**γ27**	-0.006***	0.002	**γ61**	0.020***	0.007
**γ28**	0.001***	0.000	**γ62**	-0.477***	0.055
**γ29**	-0.004***	0.001	**γ63**	-0.239***	0.005
**β2**	-0.005***	0.000	**γ64**	0.074***	0.021
**α3**	0.960***	0.242	**γ65**	0.257***	0.052
**γ31**	0.027***	0.006	**γ66**	-0.033***	0.005
**γ32**	0.074	0.048	**γ67**	1.158***	0.017
**γ33**	0.002	0.005	**γ68**	-0.005***	0.001
**γ34**	-0.042**	0.018	**γ69**	0.113***	0.005
**γ35**	-0.291***	0.046	**β6**	-0.034***	0.003
**γ36**	0.051***	0.005	**α7**	4.114***	0.284
**γ37**	-0.022	0.014	**γ71**	0.034***	0.008
**γ38**	-0.005***	0.000	**γ72**	-0.905***	0.056
**γ39**	0.072***	0.005	**γ73**	0.017***	0.006
**β3**	0.056***	0.003	**γ74**	0.013	0.021
**α4**	-1.463***	0.128	**γ75**	0.316***	0.053
**γ41**	0.026***	0.003	**γ76**	-0.073***	0.005
**γ42**	0.037	0.025	**γ77**	-0.614***	0.017
**γ43**	0.040***	0.002	**γ78**	-0.001**	0.001
**γ44**	0.023**	0.010	**γ79**	-0.106***	0.006
			**β7**	0.086***	0.003

Source: Authors’ calculation based on Pakistan PSLM information (2013–14).

***, **, and * are used for 1, 5, and 10% significance level, respectively.

#### 4.2.3 Estimated uncompensated and compensated elasticities of LA-AIDS

The uncompensated and compensated own prices elasticities of LA-AIDS are presented in Table 5. All the uncompensated and compensated own price elasticity estimates have a negative sign, clarifying the fact that a commodity’s price itself has an adverse effect on its volume demand for all six energy items, while own price elasticity for electricity is positive in both cases indicates that the electricity demand will not be decline if the price of electricity rises. Elasticities estimate results are the same for compensated and uncompensated in terms of the sign, but the magnitude is different such as the magnitude of uncompensated elasticities of natural gas, firewood, and other fuels are higher than compensated which indicates these are normal goods, while the compensated elasticity for cylinder gas and electricity is greater than uncompensated which shows that these are inferior, but for the kerosene oil and diesel the elasticities are same in magnitude which indicates that for these items there is less share of income.

**Table 5:** Own price elasticities of LA-AIDS.

Energy commodities	Uncompensated	Compensated
Fire wood	-1.44	-1.19
Kerosene oil	-10.87	-10.87
Natural gas	-1.03	-0.87
Cylinder gas	-0.08	-0.16
Diesel	-35.83	-35.83
Electricity	1.61	2.03
Other fuels	-1.58	-1.35

Source Authors’ calculation based on Pakistan PSLM information (2013–14).

Table 6 presents the uncompensated cross-price elasticity of LA-AIDS. Cross price elasticities of Firewood with respect to other six energy commodities are positive signifying gross substitute except for diesel for which firewood is complimentary due to negative elasticity. The uncompensated cross-price elasticity of kerosene oil is positive for firewood, natural gas, Cylinder gas, and Diesel and shows substitutes while w.r.t electricity and other fuels are negative and show complementary goods. Natural gas elasticities with respect to the other six energy commodities are positive signifying gross substitutes except for electricity for which natural gas is a compliment. Cylinder gas is a substitute for Kerosene oil, Diesel, other fuels, and electricity, while cylinder gas is complimentary for Fire-wood and Natural gas. Diesel is complimentary for firewood, kerosene oil, and natural gas while Diesel is a substitute for other fuels, electricity and cylinder gas. The cross-price elasticity of electricity shows that it compliments all other energy items except cylinder gas and diesel. Other fuels are a substitute for all energy commodities except electricity.

**Table 6:** The uncompensated cross-price elasticities of LA-AIDS.

**Energy Commodities**							
Fire wood		Kerosene oil	Natural gas	Cylinder gas	Diesel	Electricity	Other fuels
Fire wood		0.36	0.12	1.89	-0.34	0.06	0.09
Kerosene oil	5.23		0.66	1.36	25.33	-1.06	-6.14
Natural gas	0.67	1.03		1.86	0.53	-0.52	0.05
Cylinder gas	-0.31	0.14	-0.39		1.82	0.17	0.07
Diesel	-1.49	-1.24	-2.60	8.22		0.57	2.14
Electricity	-3.06	-0.27	-0.42	9.06	9.42		-4.43
Other fuels	0.15	0.71	0.38	0.82	0.37	-0.06	

Source: Authors’ calculation based on Pakistan PSLM information (2013–14).

The compensated cross-price elasticities of LA-AIDS are presented in Table 7. The compensated cross-price elasticities are similar to the uncompensated cross-price elasticities. All the commodities are the same for each other as substitutes and compliments except other-fuels. Other-fuels cross-price elasticities from uncompensated LA-AIDS show complementarity for electricity, but from Compensated LA-AIDS other fuels are a substitute for electricity. Electricity is also a substitute for natural gas in compensated cross-price elasticities.

**Table 7:** The compensated cross price elasticities of LA-AIDS.

**Energy Commodities**							
Fire wood		Kerosene oil	Natural gas	Cylinder gas	Diesel	Electricity	Other fuels
Fire wood		0.50	0.49	1.19	-0.39	0.29	0.48
Kerosene oil	5.24		0.68	1.33	25.33	-1.05	-6.12
Natural gas	0.78	1.09		1.55	0.51	-0.42	0.23
Cylinder gas	-0.28	0.16	-0.35		1.81	0.19	0.12
Diesel	-1.49	-1.24	-2.60	8.21		0.58	2.14
Electricity	-2.59	-0.02	0.25	7.79	9.32		-3.72
Other fuels	0.31	0.79	0.61	0.40	0.34	0.07	

Source: Authors’ calculation based on Pakistan PSLM information (2013–14).

Table 8 presents the income elasticity of LA-AIDS and demographic parameters. Estimated income elasticities for Firewood, natural gas, and other fuels are positive and greater than one implies that these items are normal and luxury, while income elasticities for kerosene oil and electricity are positive and less than one indicates that these are normal and necessity items, but Cylindrical gas and Diesel are negative which indicates that these items are inferior. Compared to the findings of [21] who reported that all fuel items are necessities for rural households while the negative sign for other fuels, Kerosene oil and firewood for urban areas shows inferior items. Household size has a positive coefficient for Firewood, Kerosene oil, and Cylinder gas. Estimate for Diesel shows that the usage of these products will increase as the size of the family rises, While the size of the family is negative for natural gas, other fuels and electricity, showing that as the size of the family rises than the consumption of these products decreases [4], the same outcomes were also recorded for the size of the family. The coefficient of appliances is significant and positive for Electricity, Natural gas, and Cylinder gas which indicates that when the number of particular appliances increases then the consumption for these items will increase, while appliances are significant but have a negative coefficient for Fire-wood, Kerosene oil and for other fuels which shows when appliances increases than consumption for these items will decrease [3], also reported the same results for appliances.

**Table 8:** LA-AIDS income elasticities and demographic parameters.

Energy commodities	Income elasticities	HH-SIZE	Appliances
Fire wood	1.04	0.008	-0.111
Kerosene oil	0.57	0.001	-0.004
Natural gas	1.50	-0.005	0.072
Cylinder gas	-2.83	0.003	0.035
Diesel	-0.22	0.000	0.001
Electricity	0.92	-0.005	0.113
Other fuels	1.58	-0.001	-0.106

Source: Authors’ calculation based on Pakistan PSLM information (2013–14).

#### 4.2.4 Comparison of income elasticities from two models

The income elasticities of various energy items from different models are presented in Table 9. The income elasticities of all the commodities are almost same except firewood as shown in graph that firewood has positive elasticity but less than one shows normal and necessity in log-log model while in LA-AIDS firewood elasticity is greater than one shows luxury item [15], also reported that firewood is normal and necessity while [6, 18] reported that firewood is inferior for urban and necessity for rural areas. Kerosene oil is normal and necessity for LA-AIDS and log-log model [6, 15], also reported that kerosene oil is normal and necessity, comparatively the magnitude of LA-AIDS elasticity is greater than double log model. Natural Gas is normal and luxury for LA-AIDS and double log model [5, 21], reported that natural gas is necessity while [6] reported that natural gas is luxury. Cylinder gas and Diesel are inferior for log-log and LA-AIDS, while [15] reported that LPG is necessity which is different from our findings. Electricity income elasticity shows that Electricity is normal and necessity item for household, similar findings are reported in [6, 21]. At the end other-fuels elasticity results are the same for two models as luxury [15], reported that other fuels are necessities while magnitude was close to unity.

**Table 9:** The income elasticities from different models.

Energy commodities	log-log	LA-AIDS
Fire-wood	0.814	1.04
Kerosene oil	0.058	0.57
Natural gas	1.038	1.50
Cylinder gas	-0.278	-2.83
Diesel	-0.291	-0.22
Other fuels	1.071	1.58
Electricity	0.404	0.92

Source: Authors’ calculation based on Pakistan PSLM information (2013–14).

The contradictions in the results of our study with literature are only for cylinder gas, this difference in the result of income elasticities may be due to the less budget share for this commodity as shown in descriptive statistics in Table 1 like the budget share for cylinder gas is 2.80 percent, very small as compared to firewood, natural gas, other fuels, and electricity.

#### 4.2.5 Summary of own price and cross-price elasticities

All the compensated own price elasticity estimates of double log model and LA- AIDS for various energy sources (fuels and other fuels) have the same negative sign, explaining the fact that the price of a commodity itself has an adverse effect on its demand for quantities, while the own-price elasticity of electricity is positive in both models. This indicates that when electricity demand is analyzed along with the demand for other energy sources, it has a positive relation with price [4], also reported the same findings for electricity. From compensated log-log model all the own-price elasticities are price inelastic except electricity [21] also reported the same findings. But from LA-AIDS model all energy sources are price elastic except natural gas and cylinder gas, while here Diesel is relatively more price elastic as compared to others [15], reported that natural gas is price elastic which is different from our findings.

Cross price elasticities from double-log model for Firewood is positive with respect to all the sources of energy, indicating that firewood is a substitute excluding natural gas and diesel where the sign is negative shows compliment for household [21] also reported similar findings. Kerosene oil is a substitute for firewood and natural gas, while kerosene oil compliment cylinder gas, diesel, other fuels, and electricity [15] also reported similar findings. Natural gas is a substitute for all other sources except other fuels and electricity [21], also reported that natural gas is substitute for kerosene oil and compliment for electricity and for other fuels [15]. Cylinder gas or LPG is a substitute for firewood, other fuels, and electricity while cylinder gas is a compliment for kerosene oil, natural gas and diesel [15], also reported that LPG is a substitute for firewood and natural gas and compliment for kerosene oil. Diesel is a compliment to all sources of energy except natural gas and electricity for which diesel is a substitute. Electricity is a compliment for firewood, natural gas and other fuels while electricity is a substitute for kerosene oil, cylinder gas and diesel [21], also reported the same findings. Other fuels are a substitute for all types of energy sources except cylinder gas and diesel for which other fuels are complement [6], reported that other fuels are a complement for all types of energy sources while [15] reported that other fuels are a substitute for firewood, kerosene oil and both type of gases.

Cross price elasticities from LA-AIDS shows that firewood is a substitute for all type of energy sources except Diesel for which firewood is a compliment [15], also reported that firewood is a substitute for all rest of energy sources. Kerosene oil is a substitute for all energy sources except other fuels and electricity for which kerosene oil is a compliment [6], also reported that kerosene oil is a compliment for electricity and other fuels while [15] reported that kerosene oil is a substitute for firewood. Natural gas is a substitute for all other energy sources which is also reported by [15], but natural gas is a complement for electricity which is also reported by [21]. Cylinder gas is a substitute for all energy sources except natural gas and firewood for which cylinder gas is a compliment. Diesel is a compliment for kerosene oil, firewood, and natural gas while Diesel is a substitute for cylinder gas, other fuels, and electricity, which is not reported by [15, 21]. Electricity is a compliment for firewood, kerosene oil, and other fuels while electricity is a substitute for natural gas, cylinder gas, and diesel [21], also reported the same findings, while [6] reported that electricity is a compliment for both type of gases. Other fuels are a substitute for all energy sources [15], also reported that other fuels are a substitute for natural gas, cylinder gas, firewood, and kerosene oil.

### 4.3 Correct functional form test for energy modeling

The functional form test i.e., C-test which is discussed in section 3.3 is applied to all the items of energy, Firewood, Kerosene oil, Natural gas, cylindrical gas, Diesel, electricity and Other fuels The compensated log-log model for all seven energy equations is tested in contradiction of a model which has same independent or similar right-hand side variables, but with different dependent variables which are budget share for various energy commodities also known as a linear approximation of almost ideal demand system (LA-AIDS).

The functional form, C-test results are presented in Table 10. The significance of λ parameter in equation (3.30) will lead to rejecting the model under the null-hypothesis. The lambda is significant for the log-log model for all energy equations excluding kerosene oil and diesel for which lambda is insignificant, so for kerosene oil and diesel we cannot reject our null that double-log model is correct, while null hypothesis that double-log model is correct is rejected for Firewood, Natural gas, cylindrical gas, other fuels and electricity. For LA-AIDS energy equations the lambda is statistically significant for kerosene oil, diesel and cylinder gas, while lambda is insignificant for the rest of the energy commodities. So we cannot reject our null that LA-AIDS model is correct for firewood, natural gas, other fuels and electricity, while null that LA-AIDS is correct is rejected for kerosene oil and diesel for which double log model was not rejected, but for cylinder gas results are inconclusive, from the findings of base paper [1] it is indicated that for inconclusive results they prefer LA-AIDS. The C-test recommends that the LA- AIDS model is not rejected for the main energy sources, as we know that budget shares for firewood, natural gas, other fuels and electricity are 96 percent for which C-test recommends the LA-AIDS. So we prefer the LA-AIDS model over double-log model. Also, the results of LA-AIDS model are according to economic theory and similar to the findings of [15] which allows to prefer LA-AIDS over double log model.

**Table 10:** C-Test for correct functional.

C-Test	Estimates	Std. Error	P-value
H_0_: The log-log model is correct			
**Fire wood**	-0.092	0.008	0.000*
**Kerosene oil**	-0.018	0.012	0.145
**Natural gas**	0.031	0.004	0.000*
**Cylinder gas**	-0.168	0.016	0.000*
**Diesel**	-0.015	0.019	0.419
**Electricity**	-0.799	0.012	0.000*
**Other fuels**	-1.145	0.004	0.000*
H_0_: The linear approximation of almost ideal demand system is correct			
**Fire wood**	0.014	0.009	0.141
**Kerosene oil**	0.027	0.008	0.001*
**Natural gas**	-0.015	0.011	0.176
**Cylinder gas**	0.203	0.006	0.000*
**Diesel**	0.011	0.002	0.000*
**Electricity**	0.002	0.003	0.513
**Other fuels**	-0.004	0.011	0.734

*Source*: *Authors’ calculation based on Pakistan PSLM information (2013–14)*. *** Shows that null hypothesis is rejected that model is correct at 1% level of significance.

### 4.4 Energy demand projections in Pakistan

Energy demand is estimated from 2015 to 2030 by using 2014 as the base year. Different energy commodity groups such as firewood, kerosene oil, natural gas, cylindrical gas, diesel, electricity and other fuels are projected.

#### 4.4.1 Total household energy consumption in Pakistan

The household energy consumption for the year 2014 is presented in Table 11. Monthly values of consumption are reported in the data set, which enables us to calculate the annual consumption and per capita consumption of the households which are shown in Table 11. Per capita household energy in the year 2014 is used as a base year for energy demand projections. All the consumption data set is taken from PSLM (2013–14). The total per capita household energy demand for fuels is observed as 243.02 kg/year which is higher than other fuels 100.57 kg/year. From fuels per capita consumption of natural gas (143.9) kg/year is higher than Firewood (97.6) kg/year, Per capita energy consumption of diesel/petrol for a generator is lowest (0.21) Ltr/year than kerosene oil 0.23 kg/year and cylinder gas 1.01 kg/year. It can be observed that per capita energy consumption for electricity (132.02 KWh) is also higher than other-fuels, because from descriptive statistics we know that 45 percent of budget shares are only for electricity, so the annual electricity demand is 15416890.32 (KWh).

**Table 11:** Total per capita household consumption in (2013–14).

Energy commodities	Monthly kg/Ltr	Annual kg/Ltr	Per Capita Consumption
Fire wood	949835.25	11398023.00	97.60
Kerosene oil	2213.41	26560.92	0.23
Natural gas	1401100.00	16813200.00	143.97
Cylinder gas	9787.83	117453.96	1.01
Diesel	2023.24	24278.88	0.21
Total fuels	-	-	243.02
Other fuels	978716.22	11744594.64	100.57
Electricity	1284740.86*	15416890.32*	132.02

Source: Authors’ calculation based on Pakistan PSLM information (2013–14).

* Shows that Electricity is in KWh.

#### 4.4.2 Population projections

The total present population and projections of population are presented in Table 12. Asian Development Bank (ADB) key indicator for the Asia and Pacific 2018 data set shows that the total population of Pakistan for the year 2014 was 188.02 million and for 2017 was 207.77 million. The average growth rate of the overall population from 2001 to 2017 was 2.36, according to ADB information, this average growth rate (2.36) is used to forecast the future population level. Based on this data, with the support of simple compound formula, we estimated the population of Pakistan from 2015 to 2030. The country’s total population is anticipated to grow from 207.77 million in 2017 to 222.80 million by 2020, 250.32 million by 2025 and 281.22 million by 2030.

**Table 12:** Total projected population from 2018 to 2030.

Years	Total Population	Years	Total Population
2014*	188.02	2023	238.92
2015*	191.71	2024	244.55
2016*	195.39	2025	250.32
2017*	207.77	2026	256.21
2018	212.66	2027	262.25
2019	217.68	2028	268.43
2020	222.80	2029	274.75
2021	228.05	2030	281.22
2022	233.43		

* Shows that total population for that year is reported in ADB data set, and next years are projected years.

#### 4.4.3 Income growth rates

The projected GDP growth rate is presented in Table 13. The projected data set is available from the key Asia and Pacific 2018 Asian Development Bank (ADB) indicator. The country’s income growth rate is anticipated to rise from 3.11 in 2014 to 3.71 in 2019, 3.90 in 2025, and 4.01 in 2030.

**Table 13:** The projected income growth rates.

Years	GDP growth rate	Years	GDP growth rate
2014	3.11	2023	3.91
2015	3.20	2024	3.93
2016	4.01	2025	3.90
2017	4.19	2026	3.92
2018	4.05	2027	3.94
2019	3.71	2028	3.96
2020	3.97	2029	3.98
2021	3.98	2030	4.01
2022	3.99		

Source: Asian development bank key indicator for the Asia and pacific 2018.

#### 4.4.4 Income elasticities from LA-AIDS model

The income elasticities of various energy commodities from LA-AIDS model are presented in Table 14. As from the results of C-test we have selected the linear approximation of almost ideal demand system (LA-AIDS) over double log model, so the income elasticities from LA-AIDS model are used for projections. It is expected that elasticities with a positive sign (normal) will increase the future demand and elasticities with a negative sign (inferior) will decrease the future level of demand for that energy item. Firewood, kerosene oil, natural gas, other fuels and electricity have a positive coefficient, so the consumption demand for these items should increase, while consumer demand for cylinder gas and diesel is expected to decrease, as the elasticities of LA-AIDS for these items indicates that the demand will decrease in future, this contradiction is may due to small budget shares for these items.

**Table 14:** The income elasticities from LA-AIDS models.

Energy commodities	Income elasticities
Fire wood	1.04
Kerosene oil	0.57
Natural gas	1.50
Cylinder gas	-2.83
Diesel	-0.22
Electricity	0.92
Other fuels	1.58

Source: Authors’ calculation based on Pakistan PSLM information (2013–14).

#### 4.4.5 Future energy demand for Pakistan

Energy demand based on various items is estimated from LA-AIDS model, to project the future level demand energy we need income elasticity which is also calculated from the models, are presented in above Table 14. Per capita household consumption, the total projected population, and the projected GDP growth rate is also shown above which will be used for projections.

#### 4.4.6 Projections based on income elasticities from LA-AIDS

The projections of per capita energy demand based on the LA-AIDS model are presented in Table 15. Projections are made under the assumption that there is constant or average growth in population, constant prices, no change in taste and preferences, and constant technology of production. Projections results of LA-AIDS for energy demand shows that the per capita demand for natural gas is expected to rise from 143.97 kg/year in 2014 to 365.87 kg/year in 2030 which is a relatively higher demand than other fuels 100.57 kg/year in 2014 to 269.32 kg/year in 2030 and firewood 97.60 kg/year in 2014 187.48 kg/year in 2030. Kerosene oil has very small positive income elasticity due to which the expected rise in demand is very small almost unchanged, as it is expected to rise from 0.23 Ltr/year in 2014 to 0.33 Ltr/year in 2030. Diesel and cylinder gas has negative elasticity and the per capita demand for these two is expected to decline from 2014 to 2030 as shown in the table but that decline in per capita demand is almost unchanged. The per capita demand for electricity at the household hold level is expected to rise from 132.02 KWh/year in 2014 to 236.32 KWh/year in 2030. So the overall energy demand for all main sources of energy is expected to double in the next decades, especially for natural gas, firewood, other fuels, and electricity.

**Table 15:** Per capita energy demand projections.

Years	Fire wood	Kerosene oil	Natural gas	Cylinder gas	Diesel	Electricity	Other fuels
2014	97.60	0.23	143.97	1.01	0.21	132.02	100.57
2018	115.08	0.25	182.16	0.62	0.20	152.91	128.94
2022	135.05	0.27	228.97	0.39	0.19	176.37	164.17
2026	157.60	0.30	285.53	0.24	0.19	202.42	207.28
2030	187.48	0.33	365.87	0.15	0.18	236.32	269.32

Source: Authors’ calculation based on Pakistan PSLM information (2013–14).

The total (kg/year) demand from two different models (double log model and LA- AIDS) and their per capita energy demand graphs for various energy items are presented in the S1 Appendix.

## 5. Conclusion

This study attempts to choose the correct functional form in energy modeling, to analyze the demand for energy in Pakistan and projections of future demand for various energy items. This analysis is based on the 2013–14 micro-level information from the measurement of social and living standards (Social & HIES) in Pakistan. The important contribution of the study is that we are selecting the correct functional form between the two alternatives by using C-test. Two alternative models, the double log model and linear approximation of the almost ideal demand system are tested that the double log model is correct under the null hypothesis for which the results of C-test indicate that lambda is statistically significant for all types of fuels and other fuels (except kerosene oil and diesel) which is against our null hypothesis, while lambda is insignificant for kerosene oil and diesel in the favor of the null hypothesis.

C-test under the null hypothesis that LA-AIDS is correct, indicates that lambda is statistically significant just for kerosene oil, diesel and cylinder for which the double log model was correct. While C-test recommends that LA-AIDS model is correct for all other main energy items like firewood, natural gas, other fuels and electricity. So the C-test allows choosing the LA-AIDS over the double log model also due to more consistent results with literature we prefer LA-AIDS model over the double log model.

Major findings of the study for various energy items are given below:

1. All the uncompensated and compensated own price elasticities have an accurate (negative) sign which shows that the price of a commodity itself has an adverse effect on its demand for quantities from both models as double log and LA- AIDS, except electricity for which own price elasticity is positive, which shows the favorable effect on its demand, as we prefer the LA-AIDS model which indicates that all the own-price elasticities are price elastic as less than negative unity (ε_ii_) excluding natural gas and cylinder gas.
2. The income elasticities of all the commodities are almost the same in both the models except firewood which is normal and necessity in log-log model while in LA-AIDS firewood is a luxury item. Kerosene oil and electricity are normal and necessity for LA-AIDS and log-log model, Natural Gas and other fuels are normal and luxury for LA-AIDS and double log model, Cylinder gas and Diesel are inferior for log-log and LA-AIDS.
3. The energy demand estimated in this empirical analysis suggests that if the population grows by 2.36 percent per year then the household per capita demand for energy products will rise over the next decade by maintaining prices constant. It is indicated that demand from households is driven by population growth and income growth.

The estimated elasticity of own prices, cross-prices and income in terms of spending (income), family size and equipment is particularly important to producers and policymakers in making investment and incentive choices. A significant part of the budget for families is for electricity, natural gas, firewood and other fuels, smaller budget shares are set down to other items such as kerosene oil, cylinder gas and diesel.

Firewood is solid fuel and the cutting process of wood for energy purposes will decrease the resources of forest and therefore the diminishing of forestry will leads to plentiful environmental harms. So this is important for governments to reflect policies that boost the use of other types of clean fuels like natural gas and cylinder gas etc. and there should be a disincentive to the use of these solid fuels as firewood. The administration should also decline the use of these solid fuels at the household level to control the numerous environmental and health problems. Due to no proper market for these solid fuels (firewood and other fuels), the authorities have restricted power to control the price of these solid fuels. So there should be a taxing policy for these solid fuels to tackle this issue, in case the government imposes a tax on firewood which results in an increase in the price of these solid fuels due to which the quantity demanded of firewood will be reduced. Interesting, when there is a tax on firewood then it would increase the demand quantity of cylinder gas (which is expected to decline in the next decades) more than as compared to natural gas. Similarly, if government subsidies on clean fuels (natural gas, cylinder gas, and electricity) for households then it would also increase the demand for these clean fuels and would reduce the consumption of solid fuels. A positive relationship exists between energy demand and household size. The predictions based on assumptions for different energy products indicate the excessive liability for producing electricity and natural gas for national demand imposed on the production industry because there is expected to high rise in demand for electricity and natural gas in the next decades. Energy policy is most important and one of the major government policies because it demonstrates national independency, sustainable economy, and society.

The results of the study show that energy prices, household income and household size all play an important role in determining the energy demand. Therefore, demand-side policies can play a vital role in decreasing the gap between energy demand and supply. Some determinants, like energy prices and household size, can be influenced by government policies. Energy prices, for example, can be influenced by the system of taxation, and household size through family planning programs. Since the presence of electricity-consuming appliances always contributes positively towards the electricity expenditure. The same evidence is empirically proved here. Air-conditioners and Freezer are the two most powerful contributors. Thus, to control or reduce the electricity demand, the use of an air conditioner and freezer must be reduced. Recently, it has become standard practice in different European countries for governments to educate households to decrease electricity consumption to conserve resources and avoid waste.

## Supporting information

S1 Appendix (DOCX)
