# Supplementary material for: Selecting correct functional form in consumption function: Analysis of energy demand at household level
Source: PLoS One. 2022 Dec 22;17(12):e0270222. doi: 10.1371/journal.pone.0270222 (PMC9778524; doi:10.1371/journal.pone.0270222)
Supplement: S1 Appendix — (DOCX) [file pone.0270222.s001.docx]

## APPENDIX

**Appendix Table 1:** *Total energy demand (Kg) projections from LA-AIDS model*

Year Fire wood Kerosene oil Natural gas Cylinder gas Diesel Electricity Other fuels

| 2014 | 11398.02 | 26.56 | 16813.20 | 117.45 | 24.28 | 15416.89 | 11744.59 |
| --- | --- | --- | --- | --- | --- | --- | --- |
| 2015 | 19332.96 | 44.40 | 28922.71 | 175.33 | 39.57 | 26057.15 | 20256.99 |
| 2016 | 20690.48 | 46.50 | 31605.98 | 154.42 | 39.90 | 27740.44 | 22222.82 |
| 2017 | 23042.40 | 50.73 | 35900.69 | 143.02 | 42.00 | 30740.18 | 25335.92 |
| 2018 | 24472.75 | 53.00 | 38739.77 | 131.38 | 42.64 | 32518.11 | 27421.07 |
| 2019 | 25668.61 | 54.98 | 41068.21 | 125.58 | 43.42 | 34016.22 | 29127.64 |
| 2020 | 27704.28 | 57.96 | 45338.14 | 109.57 | 43.93 | 36504.98 | 32292.15 |
| 2021 | 29549.10 | 60.69 | 49218.24 | 99.29 | 44.56 | 38763.04 | 35171.80 |
| 2022 | 31523.66 | 63.57 | 53446.95 | 89.90 | 45.19 | 41168.83 | 38320.75 |
| 2023 | 33355.73 | 66.27 | 57369.15 | 83.57 | 45.92 | 43404.85 | 41243.65 |
| 2024 | 35599.34 | 69.42 | 62334.61 | 75.57 | 46.58 | 46115.72 | 44963.95 |
| 2025 | 37819.79 | 72.54 | 67290.59 | 69.35 | 47.29 | 48795.21 | 48684.63 |
| 2026 | 40379.85 | 76.01 | 73156.33 | 62.63 | 47.95 | 51861.31 | 53107.92 |
| 2027 | 43111.72 | 79.64 | 79529.51 | 56.57 | 48.63 | 55118.40 | 57930.12 |
| 2028 | 46065.44 | 83.49 | 86556.41 | 50.96 | 49.31 | 58622.15 | 63266.10 |
| 2029 | 49261.46 | 87.55 | 94312.43 | 45.79 | 49.99 | 62393.85 | 69177.29 |
| 2030 | 52725.38 | 91.87 | 102890.95 | 41.03 | 50.67 | 66460.24 | 75739.77 |

*Source: Authors’ calculation based on Pakistan PSLM information (2013-14).*

**Appendix Table 2:** *Total energy demand (Kg) projections from double log model*

Year Fire wood Kerosene oil Natural gas Cylinder gas Diesel Electricity Other fuels

| 2014 | 11398.02 | 26.56 | 16813.20 | 117.45 | 24.28 | 15416.89 | 11744.59 |
| --- | --- | --- | --- | --- | --- | --- | --- |
| 2015 | 19198.90 | 43.68 | 28517.79 | 191.10 | 39.49 | 25635.92 | 19940.78 |
| 2016 | 20335.58 | 44.65 | 30519.88 | 192.16 | 39.68 | 26636.59 | 21372.74 |
| 2017 | 22426.48 | 47.60 | 33988.74 | 201.75 | 41.64 | 28845.55 | 23835.74 |
| 2018 | 23632.27 | 48.83 | 36098.27 | 204.42 | 42.17 | 29957.24 | 25343.98 |
| 2019 | 24657.63 | 50.05 | 37861.98 | 207.86 | 42.86 | 30957.09 | 26602.50 |
| 2020 | 26316.27 | 51.38 | 40864.13 | 209.66 | 43.21 | 32357.00 | 28758.56 |
| 2021 | 27824.45 | 52.72 | 43584.83 | 212.18 | 43.70 | 33660.94 | 30712.17 |
| 2022 | 29424.15 | 54.09 | 46496.83 | 214.72 | 44.20 | 35020.49 | 32805.87 |
| 2023 | 30914.79 | 55.47 | 49198.70 | 217.79 | 44.82 | 36316.13 | 34747.84 |
| 2024 | 32702.83 | 56.91 | 52507.43 | 220.37 | 45.32 | 37789.12 | 37132.44 |
| 2025 | 34468.85 | 58.37 | 55781.99 | 223.27 | 45.90 | 39249.96 | 39493.46 |
| 2026 | 36474.01 | 59.89 | 59557.32 | 225.89 | 46.41 | 40848.47 | 42221.16 |
| 2027 | 38594.78 | 61.45 | 63585.99 | 228.55 | 46.93 | 42511.50 | 45135.66 |
| 2028 | 40864.83 | 63.06 | 67941.75 | 231.18 | 47.45 | 44256.43 | 48291.31 |
| 2029 | 43296.18 | 64.70 | 72654.78 | 233.79 | 47.95 | 46087.88 | 51710.77 |
| 2030 | 45903.99 | 66.40 | 77762.80 | 236.37 | 48.46 | 48011.89 | 55422.32 |

*Source: Authors’ calculation based on Pakistan PSLM information (2013-14).*

**Appendix Figure 1:** *Projections of per capita demand for firewood*


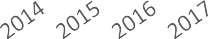

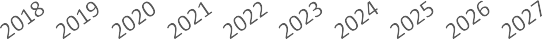

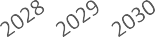


200

180

160

140

120

100

80

60

40

20

0

Fire wood

*Source: Authors’ calculation based on Pakistan PSLM information (2013-14).*

**Appendix Figure 2:** *Projections of per capita demand for kerosene oil*


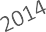

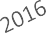

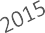

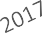

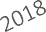

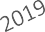

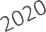

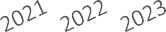

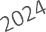

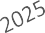

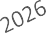

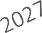

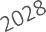

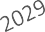

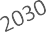


0.35

Kerosene oil

0.3

0.25

0.2

0.15

0.1

0.05

0

*Source Authors’ calculation based on Pakistan PSLM information (2013-14).*

**Appendix Figure 3:** *Projections of per capita demand for natural gas*


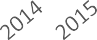

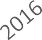

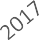

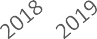

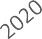

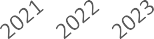

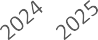

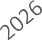

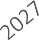

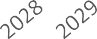

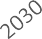


400

Natural gas

350

300

250

200

150

100

50

0

*Source: Authors’ calculation based on Pakistan PSLM information (2013-*

**Appendix Figure 4:** *Projections of per capita demand for cylinder gas*


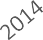

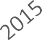

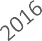

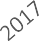

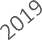

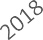

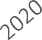

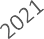

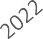

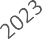

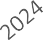

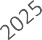

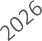

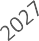

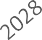

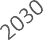

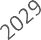


1.2

Cylinder gas

1

0.8

0.6

0.4

0.2

0

*Source: authors calculated based on Pakistan PSLM information (2013-14).*

**Appendix Figure 5:** *Projections of per capita demand for diesel*


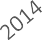

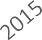

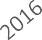

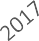

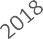

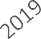

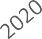

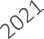

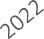

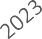

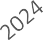

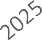

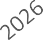

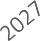

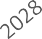

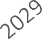

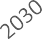


0.25

Diesel

0.2

0.15

0.1

0.05

0

*Source: authors calculated based on Pakistan PSLM information (2013-14).*

**Appendix Figure 6:** *Projections of per capita demand for electricity*


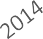

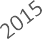

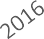

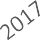

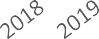

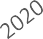

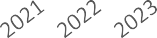

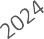

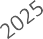

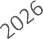

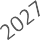

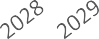

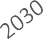


250

Electricity

200

150

100

50

0

*Source: authors calculated based on Pakistan PSLM information (2013-14).*

**Appendix Figure 7:** *Projections of per capita demand for other fuels*


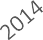

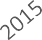

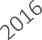

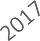

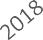

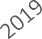

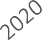

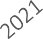

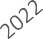

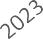

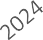

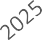

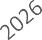

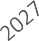

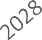

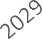

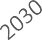


300

Other fuels

250

200

150

100

50

0

*Source: authors calculated based on Pakistan PSLM information (2013-14).*
